# Temporal Limits of Visual Motion Processing: Psychophysics and Neurophysiology

**DOI:** 10.3390/vision3010005

**Published:** 2019-01-26

**Authors:** Bart G. Borghuis, Duje Tadin, Martin J.M. Lankheet, Joseph S. Lappin, Wim A. van de Grind

**Affiliations:** 1 Department of Anatomical Sciences and Neurobiology, University of Louisville School of Medicine, Louisville, KY 40202, USA; 2 Brain and Cognitive Sciences, Center for Visual Science, Neuroscience, and Ophthalmology, University of Rochester, Rochester, NY 14627, USA; 3 Department of Animal Sciences, Wageningen University, 6700 AH Wageningen, The Netherlands; 4 Vanderbilt Vision Research Center, Vanderbilt University, Nashville, TN 37235, USA; 5 Helmholtz Institute and Department of Functional Neurobiology, Utrecht University, 3584 CH Utrecht, The Netherlands

**Keywords:** human psychophysics, apparent motion, temporal integration, cat, retina, neural coding, spike timing precision, model analysis

## Abstract

Under optimal conditions, just 3–6 ms of visual stimulation suffices for humans to see motion. Motion perception on this timescale implies that the visual system under these conditions reliably encodes, transmits, and processes neural signals with near-millisecond precision. Motivated by in vitro evidence for high temporal precision of motion signals in the primate retina, we investigated how neuronal and perceptual limits of motion encoding relate. Specifically, we examined the correspondence between the time scale at which cat retinal ganglion cells in vivo represent motion information and temporal thresholds for human motion discrimination. The timescale for motion encoding by ganglion cells ranged from 4.6 to 91 ms, and depended non-linearly on temporal frequency, but not on contrast. Human psychophysics revealed that minimal stimulus durations required for perceiving motion direction were similarly brief, 5.6–65 ms, and similarly depended on temporal frequency but, above ~10%, not on contrast. Notably, physiological and psychophysical measurements corresponded closely throughout (*r* = 0.99), despite more than a 20-fold variation in both human thresholds and optimal timescales for motion encoding in the retina. The match in absolute values of the neurophysiological and psychophysical data may be taken to indicate that from the lateral geniculate nucleus (LGN) through to the level of perception little temporal precision is lost. However, we also show that integrating responses from multiple neurons can improve temporal resolution, and this potential trade-off between spatial and temporal resolution would allow for loss of temporal resolution after the LGN. While the extent of neuronal integration cannot be determined from either our human psychophysical or neurophysiological experiments and its contribution to the measured temporal resolution is unknown, our results demonstrate a striking similarity in stimulus dependence between the temporal fidelity established in the retina and the temporal limits of human motion discrimination.

## 1. Introduction

It has been long known that the mammalian visual system is highly sensitive to motion, even when presented briefly. For example, Exner [1] reported that when humans viewed two sequentially flashed stimuli, the threshold for temporal order detection could be as short as 15 ms. Subsequent studies showed that under optimal conditions even 3–6 ms temporal-order asynchrony can be reliably discriminated [2,3,4]. Under these circumstances, the two stimuli are not perceived separately but as a single moving object (‘apparent motion’), indicating that the percept involves visual motion processing.

The middle temporal visual area (area MT, or V5) is a region of the extrastriate visual cortex in primates that has been demonstrated to be critical for motion vision [5]. Area MT has among the shortest response latencies in the extrastriate cortex [6], consistent with the observation that human reaction times are shorter for moving compared with stationary objects [7]. A short response latency is functionally meaningful because it enables a rapid response to stimulus onset, for example, during collision avoidance. But appropriate behavioral responses in a dynamic visual environment also require information about the stimulus—such as the direction of motion—to be resolved at a high temporal rate [8]. Thus, there is a benefit to encoding stimulus information on the briefest possible timescale.

Visual encoding starts in the retina, where visual transduction and signal processing within retinal neural circuits culminates in selective encoding of the visual input by the ganglion cells. Ganglion cells transmit visual information as series of action potentials (spike trains) through the optic nerve, via the lateral geniculate nucleus (LGN) of the thalamus, to the visual cortex. The majority of ganglion cells in the retinas of cats and primates signal spatio-temporal changes in luminance contrast, but do not by themselves provide information about motion direction. Instead, current working models suggest that motion vision depends on the integration of signals from multiple ganglion cells with spatially offset visual receptive fields [9,10,11,12]. This is supported by computational analysis of population macaque retinal parasol-type ganglion cell responses to a moving bar recorded in vitro, which showed that motion direction could be reconstructed from temporal correlations in the cells’ spike trains at a timescale of 10–50 ms [13,14]. Thus, the timescale at which ganglion cell spike train ensembles represent visual motion approaches the inter-spike interval. This suggests that noise variations (variability) in neuronal spike timing may limit the temporal fidelity of visual motion encoding [15,16], but to what extent they do so has remained unclear.

Variability in neuronal spike timing is apparent from trial-to-trial variations in the times at which a cell fires action potentials in response to repeated presentations of the same stimulus. Spike timing variability stems from noise in neuronal signal transduction and transmission, and the demonstrated underlying sources of this variability include quantal fluctuations in photon absorption, fluctuations in cyclic nucleotides within the photoreceptors, as well as noise in ion channels and synaptic vesicle release [17]. For several of these factors, the noise amplitude depends on stimulus parameters such as stimulus temporal frequency and luminance contrast [18,19,20,21]. Here, we postulate that if spike timing variability limits the encoding of visual motion information, then the timescale for resolving visual motion at the perceptual level should similarly depend on these stimulus parameters. In agreement with this idea, model analysis of retinal ganglion cell responses obtained from primate retinas in vitro showed that the optimal timescale for decoding retinal motion signals decreases with temporal frequency and contrast [13]. While other studies have explored the relation between encoding accuracy at the neuronal and behavioral level for chromatic [22] and orientation discrimination tasks [23], how the timescale of population motion encoding in the retina relates to the temporal limits of visual motion perception remains unclear. 

To address this, we assessed the relationship between the time scale of motion encoding in mammalian retinal ganglion cells in vivo and the temporal limits of human motion perception. We first recorded cat X- and Y-type ganglion cell spike responses to motion stimuli with a range of contrasts and temporal frequencies. We then used model analysis to compute from these responses, for each stimulus condition, the timescale at which they best represented motion information. The measured timescales approximated those reported for macaque parasol cells, supporting the assumption that the temporal precision of the retinal spike output for a subset of ganglion cell types is similar across mammals. For comparable stimuli in humans, we then measured the minimum stimulus duration required for motion direction discrimination. We found that across stimuli, the temporal limit for visual motion discrimination at the perceptual level correlated with the timescale of motion encoding at the ganglion cell level. Thus, it appears that human motion perception adheres to the temporal fidelity of visual encoding as set at the level of the retinal ganglion cells. 

## 2. Methods

### 2.1. Electrophysiological Preparation and Recordings

Extracellular single unit recordings from retinal ganglion cells and LGN cells were obtained with tungsten microelectrodes (TM33B20KT, World Precision Instruments, USA, typical impedance 2.0 MΩ at 1.0 kHz) from 19 anesthetized adult cats of either sex (3–5 kg). Surgical procedures were standard and in accordance with the guidelines of the Law on Animal Research of the Netherlands and of the Utrecht University’s Animal Care and Use Committee. 

Anesthesia was induced by ketamine hydrochloride injection (Aescoket-plus, 20 mg kg^−1^, i.m.). Following preparatory surgery, anesthesia was maintained by artificial ventilation with a mixture of 70% N_2_O-30% O_2_ and halothane (Halothaan, 0.4%–0.7%). To minimize eye movements, muscle paralysis was induced and maintained throughout the experiment by infusion of pancuronium bromide (Pavulon, 0.1 mg kg^−1^ h^−1^, i.v.). Oxygen-permeable contact lenses (+3.5 to +5 diopters, courtesy of NKL, Emmen, The Netherlands) were used to both focus the visual stimulus on the retina and protect the cornea.

LGN and optic tract recordings were obtained at approximately 10 and 20 mm below the cortical surface at Horsley-Clarke coordinates A8, L10 [24]. Action potentials from single cells were detected with a window discriminator (BAK Electronics Inc.) and digitized at 2.0kHz (PCI 1200, National Instruments) for on-line analysis and storage (Apple Macintosh G4 computer, custom-written software). 

### 2.2. Visual Stimulation 

Stimuli for electrophysiology experiments were computer-generated (ATI rage graphics card, Macintosh G4 computer, custom-written software), presented on a linearized 19”, 100 Hz CRT monitor (Sony Trinitron Multiscan 400PS) at 57 cm from the optic node and centered on the receptive field of the cell under study. Mean luminance was 54 cd·m^−2^. For those cells (<15%) that showed significant response modulation to the 100Hz refresh rate of the monitor [25], the frame rate was increased to 120Hz. 

For each cell, spatial and temporal tuning curves were measured using drifting sinusoidal gratings (spatial frequency 0.1–4.0 cycles deg^−1^, temporal frequency 0.5–50 Hz). Cells were classified as X or Y on the basis of a null-test [26]. Responses to twenty repeats of a 3 second presentation of drifting sine wave gratings were used for the model analysis. Sinusoidal gratings fully covered the receptive field and spatial frequency was optimized for each cell. While the range of spatial frequencies used to determine each cell’s tuning function spanned a 40-fold range, tuning functions of X- and Y-type cells were highly stereotyped and the range of optimal spatial frequencies within a type was narrow: X-type cells concentrated at relatively high, and Y-type cells at relatively low spatial frequencies, as expected. There was an approximately 8-fold difference in optimal frequency between the two types, but a less than 2-fold difference within each type. Thus, across each type’s sampled population, speed was approximately proportional with temporal frequency. Because the stimulus was cyclic, and because early coding of motion direction as a function of temporal frequency is well documented ([27,28]), data are presented throughout as a function of temporal frequency. Temporal frequency and luminance contrast were varied (0.5–16 Hz and from 10–70% Michelson contrast, respectively). A stimulus block consisted of 6 temporal frequencies and 7 contrasts, resulting in 42 stimuli presented in random order. Data presented in this study were obtained from cells with receptive fields located within the central 15 degrees of the visual field. Only single unit recordings that were stable during at least 20 repeats of the stimulus block and showed significant response modulation to the high contrast stimuli were accepted for analysis. 

### 2.3. Psychophysics

Stimuli for human psychophysics experiments were computer-generated using Matlab (The Mathworks; Natick, MA), the Psychophysics Toolbox [29] and Video Toolbox [30], and shown on a linearized monitor (800 × 600 pixels, 200 Hz). We used a bit stealing technique [31] to expand gray-scale resolution from 256 to 768 levels. To obtain a 200 Hz refresh rate, we used a high-speed PROCALIX monitor (Totoku, Irving, TX) driven by a MP960 graphics card (VillageTronic, Berlin, Germany). Viewing was binocular at 83 cm (yielding 2 × 2 arcmin per pixel). Luminance of the gray screen background was 41.1 cd/m^2^. Three observers participated in the experiment (first and second authors and a naïve observer). All procedures complied with institutionally reviewed guidelines for human subjects at Vanderbilt University and University of Rochester and all subjects provided written informed content.

Stimuli were vertically oriented Gabor patches, comprising a drifting vertical sine grating windowed by a stationary two-dimensional Gaussian envelope (2σ width = 20 arcmin, spatial frequency = 3 cycles/deg, starting phase randomized). Gabor contrast was modulated by a temporal Gaussian envelope. Peak Gabor contrast and temporal frequency were varied in a 7 × 5 design (0.5–32 Hz and 5%–80%, respectively). The observers’ task was to discriminate motion (left vs. right) of a briefly presented Gabor patch. Duration thresholds [32,33,34] were estimated using two interleaved QUEST staircases [35], where staircases adjusted the standard deviation of the temporal Gaussian envelope and converged to 82% correct. Duration was defined as 2σ width of the Gaussian envelope. The entire set of 35 conditions was repeated four times in random order. This yielded eight threshold estimates per condition, of which the first two thresholds were discarded as practice. Trials were self-paced. Each trial began with a key-press, followed by a stimulus 350 ms later. Feedback was provided.

Given that we were expecting very brief motion direction thresholds (especially for high temporal frequency conditions), we paid close attention to determining the lower limit of temporal stimulus duration that we could accurately present and measure. Stimuli were displayed on a 200 Hz monitor by discrete sampling of the temporal Gaussian waveform every 5 ms, while ensuring that the middle sample always contained the peak of the Gaussian [34]. For example, a Gabor patch presented in a temporal Gaussian window with 2σ = 5.6 ms (our lowest threshold: 32 Hz motion, 80% peak contrast) would be shown in 3 video frames displaying 20.1%, 100%, and 20.1% of the peak contrast (see Figure 7 for another example). To test for possible floor effects at the highest stimulus temporal frequency (32 Hz) we conducted two control experiments. First, using the same display system as in the main experiment, we measured duration thresholds for 8, 16, and 32 Hz motion at 100 Hz and 200 Hz frame rates. Substantially lower thresholds for 8 and 16 Hz stimulus presented at 200 Hz would indicate deleterious under-sampling of the Gaussian waveform at 100 Hz. Respective thresholds for 8 and 16 Hz motion were 7.9% and 8.3% lower at 200 Hz than at 100 Hz frame rate, likely indicating the effects of higher fidelity motion representation at 200 Hz. In contrast, the threshold for 32 Hz motion was 28% lower at 200 Hz, indicating a floor effect for 32 Hz motion at 100 Hz frame rate. Based on these measurements, we can assert that our setup is adequate for measuring the 32 Hz stimulus presented at a frame rate of 200 Hz. Second, we used a custom 360 Hz display system [36] and measured discrimination thresholds for 32 Hz motion presented at 90 Hz, 180 Hz and 360 Hz frame rates. Averaged over 3 subjects, the thresholds at 90 Hz, 180 Hz and 360 Hz were 5.55, 4.51, and 4.27 ms, respectively. These results accord strongly with our initial measurements, showing a modest increase in thresholds going from 360 to 180 Hz (5.67% increase) and a larger, 30%, increase at 90 Hz (F_2,4_ = 8.3, *p* = 0.038; with a significant difference between 90 Hz and 360 Hz, *p* < 0.05, Tukey HSD; individual subject analysis, all F_2,6_ > 6.8, all *p* < 0.029; all subjects exhibiting significant differences between 90 Hz and 360 Hz (*p* < 0.05), 2/3 subjects also exhibiting significant difference between 90 Hz and 180 Hz (*p* < 0.01), no significant differences between 360 Hz and 180 Hz). 

### 2.4. Model Analysis 

The aim of our model analysis was to determine the timescale at which information about the stimulus is represented in the spike response of the sampled neuron types. We measured this timescale by comparing the total correlation following temporal integration of pairs of recorded spike trains with the total correlation between pairs of ‘shuffled‘ spike trains. These shuffled spike trains were constructed from the recorded spike trains by randomly rearranging their spike time intervals, to eliminate stimulus-dependent temporal structure while maintaining first-order statistics including mean rate and inter-spike interval distribution. We defined the time constant for temporal integration time constant that maximized the difference between original vs. shuffled spike trains peaks as the ‘optimal integration time’, i.e., the timescale at which the neuronal response best represented the stimulus. 

The input signals in our model were the recorded retinal spike trains. Stimulus-dependent temporal structure in these spike trains is determined by the cell’s time-varying response rate as well as the magnitude of stimulus-independent noise variations (variability) in the temporal spike patterns. If variability in the temporal spike pattern is large, then the correlation between responses to repeated stimulus presentations will be small. Because the detection of visual motion must rely on stimulus-dependent temporal structure of the spike response, variability in a cell’s temporal spike pattern should limit motion detection. Stimulus independent response variability can be countered by integrating the spike trains over a finite time window to increase temporal overlap. But temporal integration comes at a cost of increasing the timescale at which motion may be resolved. To assess this trade-off, for the recorded ganglion cell responses we measured how the integration time that maximized stimulus-dependent temporal structure varied with stimulus contrast and temporal frequency—parameters expected to affect ganglion cell spike response variability. 

To this end, we measured cross-correlations between pairwise combinations of spike trains recorded from a single ganglion cell for a range of integration times, evoked by repeated presentation of the same visual stimulus (minimum of 20 stimulus repeats; n = 33 retinal X cells, 4 retinal Y cells, and 20 LGN X cells). We compared the cross-correlation values against those obtained from shuffled versions of the same spike responses, constructed by randomly rearranging their inter-spike intervals to remove their stimulus-dependent temporal structure while maintaining first-order statistics of the response (mean rate and inter-spike interval distribution). The optimal integration time was defined as the integration time constant where the difference between cross-correlation functions of the recorded and shuffled spike responses peaked. The details of this analysis were as follows. 

Input to the model was a set of recorded spike trains *s_i_(t)*, *n* ≥ 20,

(1) $$s_{i}\left(t\right)=\sum_{\left\{t_{j}\right\}}\delta \left(t-t_{j}\right);\;i=1,\ldots ,n.$$

Spike trains were passed through a first order filter with time constant *τ*, and normalized for *τ*, adding an exponential tail with an integral of 1 to each spike,

(2) xi(t,τ)=si(t)∗e−1ττ

From this set, pairs of spike trains were multiplied, integrated and normalized to the integral of the first spike train,

(3) $$y\left(\tau \right)=\frac{2\cdot \sum_{k=1}^{n}\sum_{m=k+1}^{n}\int_{0}^{T}dt\cdot x_{k}\left(t,\tau \right)\cdot x_{m}\left(t,\tau \right)}{\left(n-1\right)\sum_{k=1}^{n}\int_{0}^{T}dt\cdot x_{k}{\left(t,\tau \right)}^{2}}$$

This operation was performed for a series of τ ranging from 1–500 ms resulting in *y(τ)*. Spike trains *s_i_(t)* were then shuffled by redistributing the inter-spike intervals in each spike train. This yields spike trains *s_i_’(t)* that have identical mean firing rates, yet lack all stimulus-related temporal structure. Repeated for shuffled spike trains *s_i_’(t)*, the same procedure results in *y’(τ)*, which was used as a measure of chance-level coincidence between spikes in the two the spike trains given the mean spike rate. The difference function *C(τ)* describes the specific contribution of the temporal structure of the input spike trains to the coincidence detected by the hypothetical correlator unit.

(4) C(τ)=y(τ)−y'(τ)

This analysis incorporates low-pass filtering (temporal integration) and cross-correlation of neuronal responses to assess the predominant timescale at which information about the moving stimulus is represented in the spike response. For each cell and stimulus condition, all possible response pairs (180 minimum) were used in the simulations. τ_opt_ was calculated by averaging the results from each spike train pair. The procedures followed were the closest possible numerical approximation (time base 0.5 ms) to the equations presented here.

## 3. Results

### 3.1. Electrophysiology and Modeling

**Figure 1:**
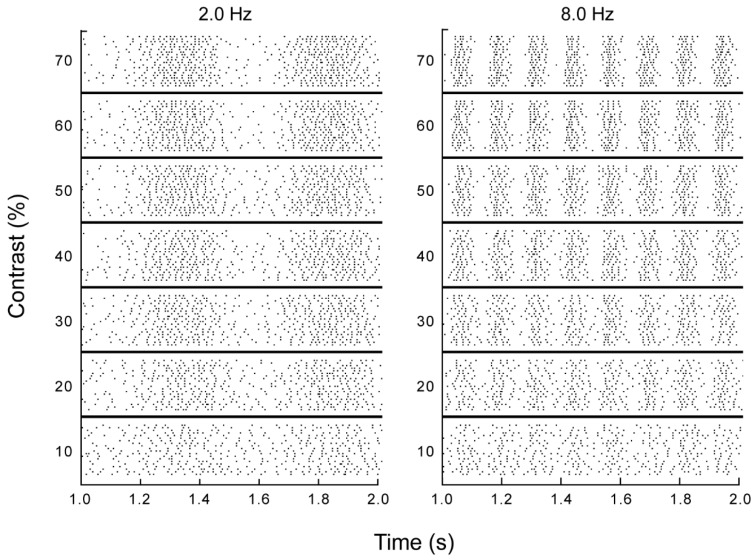
Retinal ganglion cell responses to drifting sinusoidal grating stimuli. Raster plot of a 1 second section of the response of a single retinal ganglion cell to drifting sinusoidal grating stimuli, varying in contrast (10%–70%) and temporal frequency (left 2.0; right 8.0 Hz). Each dot in the display represents a spike. Each line represents the response to a single presentation of the stimulus. Stimuli were presented randomly interleaved, and repeated a minimum of 20 times.

We recorded extracellular spike responses to repeated presentations of drifting sine wave gratings from 37 retinal ganglion cells (n = 33 X-type, 4-Y type) from the optic tract and 20 visual relay cells (all X-type) from the lateral geniculate nucleus of anesthetized cats in vivo. Spatial frequency was optimized for each cell, and temporal frequency and luminance contrast were varied (0.5–16 Hz; 10%–70%). Increasing contrast increased the modulation amplitude of a cell’s firing rate, as expected (Figure 1). It is readily apparent from these responses that the temporal structure of the neuronal response is challenged by stimulus-independent noise variations in the timing of the action potentials that make up the response. For example, at a temporal frequency of 2 Hz the stimulus is readily resolved from the time varying spike rate at a timescale of a second, whereas the times of occurrence of spikes on consecutive presentations of the same stimulus varied by several milliseconds, demonstrating that this stimulus could not be reliably resolved at a millisecond timescale. Our first goal was to assess the timescale of visual encoding in the presence of noise, and to determine how this timescale depended on the varied stimulus parameters, contrast and temporal frequency. 

**Figure 2:**
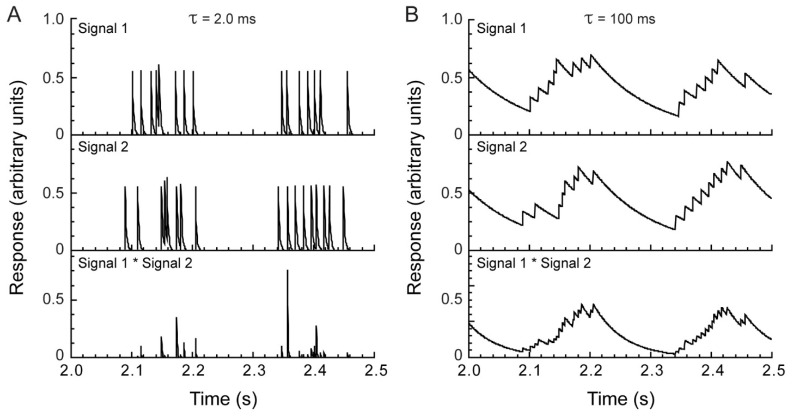
Temporal integration improves correlation detection. (**A**) A short integration time (2 ms) yielded little overlap between two spike responses evoked by repeated presentation of the same stimulus (top, middle). Only highly coincident spikes (temporal deviation < ~4 ms) resulted in non-zero output (bottom). (**B**) A long integration time (100 ms) yielded substantial overlap between the two input signals and resulted in a strong output signal (bottom).

We used model analysis to determine the predominant timescale at which spike responses represent information about the visual motion stimuli. Spike trains were first low-pass filtered with a leaky integrator-type filter characterized by a time constant τ (Figure 2A) and then cross-multiplied to determine the amount of temporal correlation. This choice of filter was motivated by its simplicity and physiological relevance, as for a range of values of τ, the exponential tail can be interpreted as a first-order description of a receiving neuron’s postsynaptic potential [37]. Low-pass filtering transformed the spike train from a temporal point process with a time-varying rate into a continuous signal—a series of superimposed pulses with exponentially decaying tails. 

**Figure 3:**
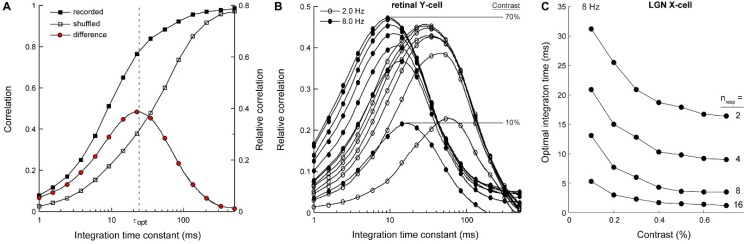
Computing the optimal integration time across stimulus conditions. (**A**) From the difference between the correlation curve for the recorded (solid squares) and shuffled spike trains (open squares) we obtained a relative correlation curve (red circles; see Model for details). We defined the optimal integration time (τ_opt_) as the time constant where the relative correlation curve peaks. At this integration time, the correlator best extracts motion information from the temporal structure of the input spike trains. Optimal integration times were computed in Matlab, following cubic spline interpolation of the 15 data points. (**B**) Relative correlation curves based on the data partially displayed in Figure 1 and Figure 3A. τ_opt_ decreases with increasing temporal frequency. Contrast (10–70%) determined total correlation (peak height), but above about 10% had little effect on τ_opt_. (**C**) τ_opt_ for a LGN X-cell computed by combining increasing numbers of spike responses. For instances 4–16, spike responses were combined additively prior to temporal filtering and pairwise cross-correlation.

Due to variability in spike timing, spikes in the two input spike trains rarely occurred within the same 0.5 ms spike acquisition time bin. Thus, for very small values of τ (<1 ms), cross-multiplication of the two spike trains gave a near-zero output signal (Figure 2B). For large values of τ, on the other hand, the correlator was largely insensitive to the timing of individual spikes, and its output reflected the mean difference in firing rate [37], which was normalized in the model, so that for large τ, the signal correlation approached unity. Between these two extremes, the correlation grew monotonically with the value of the time constant (Figure 3).

To determine how much motion information was carried by the temporal structure of the spike trains, the procedure was repeated after randomly shuffling the inter-spike intervals in each spike train. This eliminated the temporal structure while preserving response statistics such as mean firing rate and the inter-spike interval histogram. Again, correlation as a function of τ was a monotonic function (Figure 3). However, shuffling shifted the curve towards larger τ, indicating that to obtain the same level of correlation now required a longer integration time. 

The shift shows that by discarding the temporal structure of the spike trains, motion information was lost. Exactly how much information was lost is expressed by the difference between the original curve and the shuffled response curve (Figure 3). This difference function peaked at an intermediate value of τ, about 23 ms in this example. At this integration time, the motion detector maximally extracts motion information from the temporal structure of the input spike trains. We defined this value of τ as the optimal integration time (τ_opt_). Since motion detection may depend on integration of >2 spike responses, we also tested how τ_opt_ varied as a function of the number of combined spike responses. We found that combining additional spike responses prior to pairwise cross-correlation decreased τ_opt_ ~proportional to the number of integrated spike responses. However, increasing the number of integrated responses did not alter the dependence of τ_opt_ on stimulus contrast (Figure 3C) and temporal frequency (data not shown). In subsequent analyses, we used pairwise integration of two responses only, exhaustively sampled from the set of responses recorded from each cell (n = 20 repeats; 180 unique pairs).

**Figure 4:**
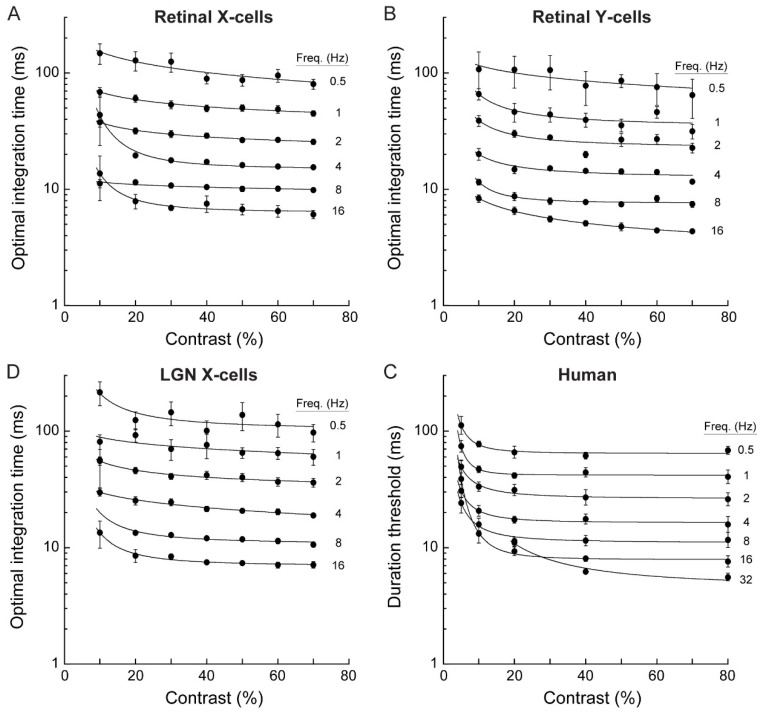
Optimal integration times and duration thresholds decreased with increasing temporal frequency but changed little with contrast. (**A–C**) Optimal integration times for all combinations of stimulus temporal frequency and contrast for 33 retinal X cells, 4 retinal Y cells, and 20 LGN X cells. Optimal integration time systematically decreased with increasing temporal frequency, but was largely independent of contrast above about 10%. Error bars show mean ± SEM. (**D**) Minimal presentation duration required for human observers to discriminate motion direction as a function of stimulus temporal frequency and contrast. The duration threshold decreased with temporal frequency of the sinewave grating. Above about 10%, the duration threshold was largely independent of stimulus contrast. Error bars show mean ± SEM for four subjects.

We found that temporal correlations between spike responses increased with increasing stimulus contrast, but above about 10%, contrast had very little effect on the optimal integration time (Figure 4A–C). This was surprising, considering the large apparent effect of contrast on spike timing variability (Figure 1). Instead, optimal integration times depended strongly on temporal frequency; increasing temporal frequency caused correlation curves to peak at shorter integration times. This effect was robust (~20-fold change across the presented frequency range) and was observed for all recorded cell types (retinal X, Y and LGN X-cells; Figure 4). 

Retinal Y cells had the shortest optimal integration time, ranging from 79 ms at 0.5 Hz to 4.6 ms at 16 Hz (n = 4), indicating that these cells had the highest temporal fidelity. The optimal integration time for retinal X cells was slightly longer, ranging from 91 ms at 0.5 Hz to 6.6 ms at 16 Hz (n = 33). The optimal integration time for LGN X cells was slightly longer again, ranging from 113 ms at 0.5 Hz to 7.3 ms at 16 Hz. Optimal integration times for LGN X cell responses were on average 26.3% ± 13% longer than those for retinal X cells, suggesting some loss of temporal precision at the LGN-relay. Optimal integration times for Y-type retinal ganglion cells were on average 18.4 ± 7.8 ms shorter than for retinal X cell responses, demonstrating greater temporal precision in Y-type cells. 

### 3.2. Psychophysics

Across stimulus parameters, the time constant that maximized motion encoding in cat retinas (above) approximated the values reported from primate retinas [14], indicating that temporal fidelity may generalize across higher mammals, including humans. If the optimal time constant for temporal integration reflects the timescale at which retinal spike trains represent motion information, then presenting motion stimuli at shorter timescales should impair cortical motion processing. Impaired cortical motion processing, in turn, should impair psychophysical performance in a motion discrimination task. To test this, we next measured how human motion discrimination depends on stimulus duration, and compared the minimum exposure duration required for resolving motion direction at the perceptual level, with the optimal integration times computed from the output of the retina and LGN. 

Duration thresholds [32] were measured for a direction discrimination task in which observers discriminated motion (left vs. right) of a foveal Gabor stimulus. Stimulus size (0.33 deg at 2σ of the spatial Gaussian envelope) approximated the foveal V1 receptive field size (0.25 deg; [38]), small enough to avoid contrast dependent center-surround interactions reported for larger moving stimuli [39]. Spatial frequency was optimized for the human fovea (3.0 c/deg; [40]). Contrast and temporal frequency—parameters known to affect motion perception (e.g., [41,42,43,44,45])—were systematically varied. 

**Figure 5:**
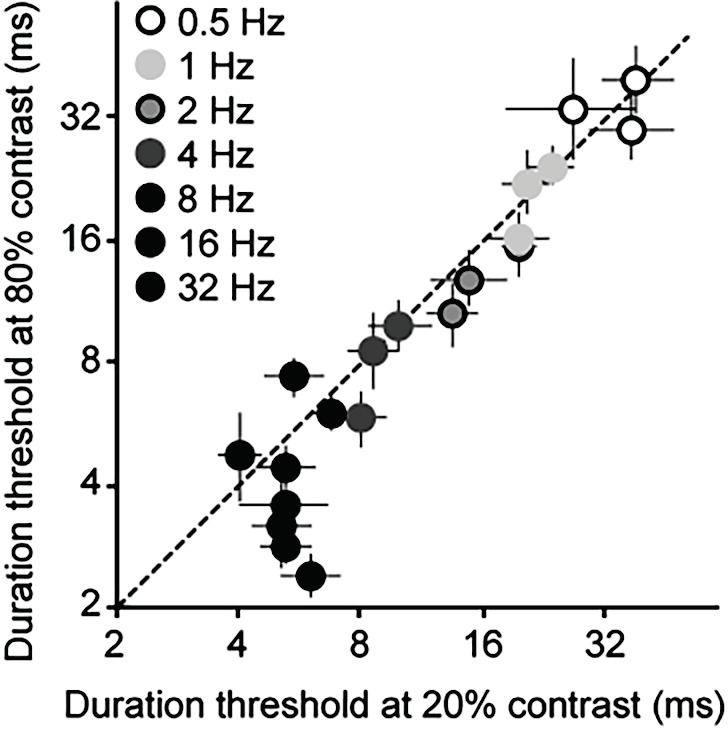
Effects of contrast on duration thresholds. Thresholds at 20% contrast are plotted against those at 80% contrast for a range of temporal frequencies. Data are shown for individual subjects, with error bars showing 95% confidence intervals.

Psychophysical duration thresholds were very short, ranging from 5.6 ms at the highest temporal frequency tested (32 Hz) to about 65 ms at 0.5 Hz (Figure 4D). Across stimuli, duration thresholds were comparable to the optimal integration times computed from the responses of retinal X, Y and LGN cells (Figure 4A–D). Optimal integration times computed from the electrophysiological data and human duration thresholds both showed a robust dependence on temporal frequency that was largely independent of stimulus contrast. For human data, thresholds increased dramatically at combinations of low contrast (< ~10%) and high temporal frequency (16–32 Hz). Because contrast sensitivity is known to decline strongly at high temporal frequencies [46] these increased thresholds likely reflect impaired stimulus detection. The key feature of the data is the apparent independence of thresholds on stimulus contrast for mid and high contrasts (Figure 4D). Indeed, when thresholds at 20% contrast are plotted against those at 80% contrast, results cluster around the identity line for all but the fastest stimuli, which exhibit lower thresholds at 80% contrast (Figure 5). 

**Figure 6:**
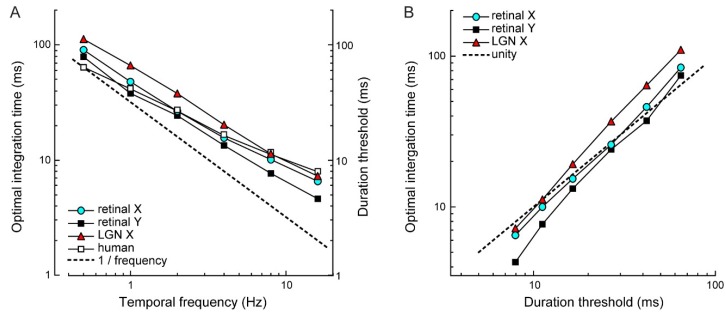
Similar stimulus dependence of optimal integration times and human duration thresholds. (**A**) Optimal integration times, averaged across responses to 40%–70% contrast stimuli, decreased with increasing temporal frequency. A similar decrease was observed for human duration thresholds. The slope of each curve deviates systematically from 1/frequency (dotted line). That is, both optimal integration time and duration threshold do not simply reflect detection of a fixed stimulus displacement distance. (**B**) Optimal integration times are comparable to human duration thresholds, except at the highest temporal frequencies, where optimal integration times for retinal Y cells are shorter than the duration threshold, i.e., their temporal fidelity exceeds psychophysical performance.

To examine the correspondence between the psychophysical and physiological results, we calculated asymptotic values of duration thresholds and τ*_opt_* estimates at each temporal frequency (Figure 6A). Asymptotic duration thresholds and τ*_opt_* estimates for different cell types were closely correlated (human vs. retinal X, r = 0.99; human vs. retinal Y, r = 0.98; human vs. LGN X, r = 0.99; all *p* < 0.0001; Figure 6B). Thus, duration thresholds and optimal integration times show the same quantitative dependence on temporal frequency.

A critical feature of our results is that the observed dependency on temporal frequency cannot be explained by the time it takes stimuli to cover a fixed proportion of its temporal cycle, or a fixed displacement distance. These, arguably less interesting explanations, would lead to proportionally shorter thresholds with increasing temporal frequency. This was not the case. Expressed as a fraction of the stimulus cycle, human duration thresholds range from as little as 1/30 of a cycle (~ 0.7 arcmin) at 0.5 Hz to 1/5 of a cycle (4 arcmin) at 32 Hz. This six-fold increase in the threshold displacement rules out the hypothesis that the threshold requires a fixed displacement of the stimulus cycle. Analogously, if optimal integration times simply reflect the linear interaction between the sine wave stimulus and the low-pass filter of the detector model, we should expect a slope of 1/frequency (Figure 6A, dotted line). For all curves, the slope is significantly shallower (paired t-test; retinal X: *p* < 0.01; retinal Y: *p* = 0.087; LGN X: *p* = 0.016; human: *p* < 0.01) indicating that a proportionally smaller stimulus period is required for direction discrimination at higher temporal frequencies. Thus, the relationship between temporal frequency and both duration thresholds and τ*_opt_* is non-linear. A likely explanation is that at high temporal frequencies, temporal deviations in spike timing and unreliable spike generation—where a cell may skip its spike response to a stimulus period—become predominant in the response’s temporal structure, and disproportionately increase the optimal integration time compared with lower temporal frequencies.

## 4. Discussion

For a range of stimulus parameters, we measured: (1) the timescale at which a motion detector model optimally detects motion from retinal ganglion cell responses, and (2) the temporal threshold of human motion perception. The timescales of motion encoding that we computed for response pairs recorded from cats in vivo closely matched reported values obtained from macaque retina in vitro [14]. We found that across conditions, both the physiological optimal integration time and the psychophysical temporal limit changed more than 20-fold. This change was non-linear with changes in temporal frequency and contrast. Importantly, over the entire range of stimulus parameters, the two measurements were comparable: human duration thresholds and optimal integration times showed a corresponding dependency on temporal frequency with negligible influence of contrast above ~10% (Figure 4 and Figure 6). 

This pattern of results is consistent with the hypothesis that spike timing variability, a co-determining factor of the optimal integration time, is an important factor limiting the temporal resolution of motion processing. Our interpretation is that spike timing variability sets the shortest sequence of spikes that needs to be analyzed by a motion detector to reliably signal motion, and that this temporal integration limits the minimum stimulus exposure required for an observer to perceive motion direction. Note that the brief integration times reported here are categorically different from the considerably longer temporal summation of motion signal investigated elsewhere (e.g., [41]), which is thought to primarily reflect integration of neural signals at stages downstream from motion detection. Our results show that the temporal limits of human motion perception closely adhere to the timescale at which motion information is best extracted from neuronal responses at the level of the retina and LGN, suggesting the high temporal fidelity of the retinal input is maintained and utilized in the visual cortex.

### 4.1. Comparison to Other Reports of Motion Acuity 

Our lowest threshold (5.6 ms at 32 Hz) is comparable to the shortest temporal order judgments reported in the literature, 3–6 ms [2,3,4]. It should be noted, however, that the stimuli used in previous studies demonstrating hyperacuity for temporal order judgments were lines or circles, sequentially flashed at two spatially separate locations. In each of these studies, total stimulus duration exceeded 10 ms. Our results show that even briefer presentations suffice: drifting Gabor stimuli that are narrowband in both space and time give similar temporal hyperacuity. Interestingly, psychophysical reports of temporal hyperacuity in vision are generally restricted to stimuli with motion cues. When such cues are removed, temporal acuity worsens to about 20–30 ms [47,48], which is comparable to the general temporal resolution of human vision [49]. This suggests that the motion system has access to temporal fidelity that is not available to other visual sub-modalities.

**Figure 7:**
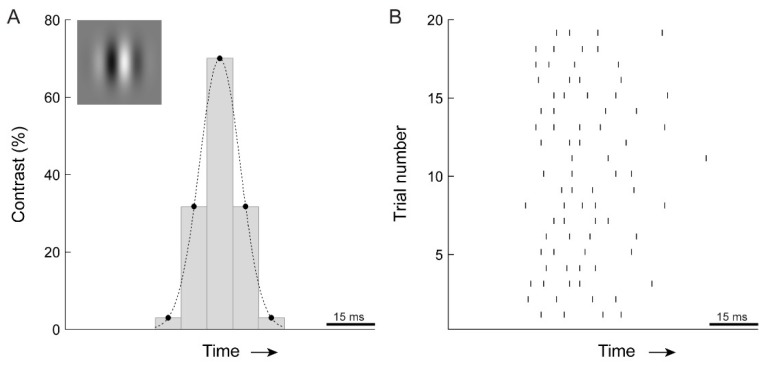
Example showing cat retinal spike response to a stimulus that is detectable by humans. Human observers were asked to discriminate the motion direction of a drifting sine wave grating (16 Hz) in a spatial Gaussian envelope (Gabor patch, top left). (**A**) The contrast of the Gabor patch was Gaussian modulated in time. Presented at 200 Hz, this paradigm allowed very brief presentations of stimulus motion. The example shows the discrete sampling of contrast values (σ = 7.9 ms, 70% contrast). (**B**) To a drifting sine wave (16 Hz) of the same contrast, a cat retinal ganglion cell fires ~4 spikes. Our analyses show that without additional loss of spike timing precision at subsequent signaling stages, spike responses such as these allow direction discrimination on a timescale similar to those measured in the human psychophysics experiments. The analyses also showed that integrating responses from additional neurons shortens this timescale. Thus, there is a potential trade-off where loss in spike timing precision at subsequent signaling stages may be counteracted by integration over additional cells. While the actual number of neurons integrated in our motion discrimination task is not known, and cannot be resolved from our data, it is highly likely that even the percept of a small motion stimulus involves multiple cells. This would imply some loss of precision of neuronal responses after the LGN.

The brief psychophysical thresholds measured here and in earlier studies (less than 10 ms) imply that motion direction can be computed from just a few spikes per cell. To illustrate this, Figure 7 shows, side-by-side, the 16 Hz stimulus successfully discriminated by human observers (7.9 ms threshold; Figure 6) and a retinal X cell’s response to one period of a drifting sine wave. A cell typically fired 3 to 4 spikes during the time approximating the psychophysical stimulus presentation. For 32 Hz motion, which yielded the 5.6 ms psychophysical threshold, the number of spikes was even lower. This suggests that for optimal stimuli, motion direction can be computed from just a few spikes per retinal input. Such estimates, of course, are likely to be noisy but can be improved by integrating responses from additional neurons [14]. This would establish a trade-off between temporal acuity and spatial acuity.

### 4.2. Comparing Electrophysiology to Psychophysics 

This study connects results derived from neurophysiological recordings in an in vivo animal model with human psychophysical data—a link that should be treated with care [50]. To do so, it is important to consider the underlying assumptions along with the experimental choices that were made, to determine the extent to which the comparison of neurophysiological and psychophysical results was justified and meaningful. 

First, we considered the assumptions behind the neurophysiological recordings and accompanying modeling. The relevance of these results depends on: (1) the functional significance of the optimal integration times, (2) the implications of mimicking pairs of cells with two responses from the same cell, and (3) the homology of temporal limits in motion processing between cats and primates, including humans.

### 4.3. Significance of the Optimal Integration Time 

Our model analysis of ganglion cell spike trains yielded brief optimal integration times, and earlier work showed feasibility of decoding spike trains on a similarly short timescale [13]. However, it is not guaranteed that the timescale over which the motion system integrates its inputs is, in fact, optimized for temporal resolution. Indeed, a shorter-than-optimal integration time could result in attenuated, but nonetheless significant detection (Figure 3), establishing a trade-off between signal-to-noise ratio, i.e., ‘certainty’, and temporal resolution. Indeed, the motion cortex may sacrifice signal-to-noise ratio to increase temporal resolution: for example, longer, sub-optimal temporal integration could compensate for an apparent loss of temporal resolution at the LGN X cell relay (Figure 6). Thus, while, τ_opt_ represents the timescale that would enable a cell to maximize signal-to-noise ratio of the motion-evoked response, the actual parameters used in cortical motion computation remain unclear. Finally, whether the timescale for encoding motion within a given neural circuit is fixed, or whether the same circuit can adapt its integration time depending on the task demands, remains to be determined. 

### 4.4. Use of Single Cells to Assess Correlation Detection

The premise of our computational analysis was that the detection of visual motion direction relies on the detection of temporal correlations in the motion-evoked neuronal response. The analysis of stimulus-dependent temporal structure in the recorded responses was based on pairwise cross-correlation of spike trains following temporal low-pass filtering (Figure 2A), using two responses from the same cell, exhaustively sampled from the recorded dataset of 20 stimulus repeats. Because the spike trains used to assess correlations in temporal structure were generated by the same cell, our analysis gives an upper bound to the correlation coefficient and lower bound to the optimal integration time. Combining responses from increasingly dissimilar cells should reduce response correlations and increase the optimal integration time. We found that, as expected from central limit theory, and as reported in macaques previously [14], combining increasing numbers of spike trains increased temporal correlations and lowered the optimal integration time (Figure 3C). However, the specific pattern of its dependence on stimulus contrast and temporal frequency remained unchanged. 

### 4.5. Species Differences 

We compared our results obtained from cats, in vivo, with those of Chichilnisky and Kalmar [13], who employed a bi-local detector model to compute optimal timescales for motion discrimination from ganglion cell responses recorded in the macaque retina, in vitro. For comparable stimuli, our estimates of optimal integration times from cat X- and Y-cells closely matched the ‘optimal temporal filter widths’ reported for macaque parasol cells [13]. The optimal integration time for a cat retinal ganglion cell responding to a sine wave drifting at 14 deg sec^−1^ was 12 ms. For macaque parasol cells responding to a bar also moving at 14 deg sec^−1^, the optimal timescale reported by Chichilnisky and Kalmar [13] was 13 ms. Thus, in terms of the timescale of motion encoding, cat X and Y, and macaque parasol cells appear comparable.

The similarity between response temporal fidelity in cat and primate retinas is perhaps not surprising because variability in spike rate and spike timing is likely to be similar across these species. Response variability at the level of the retina depends on four key factors: neural noise amplitude, contrast sensitivity, refractory period, and peak firing rate. Because these are fundamental properties shared among equivalent cell types (e.g., cat Y and primate parasol), one would not expect major differences between them, and none have been reported. On the contrary, for a spatio-temporal white noise stimulus, cat and macaque retinal responses are reportedly highly similar (cat: [51,52,53]; macaque: [21,54]). Thus, our measurements agree with the established similarities of cat ganglion cell and macaque parasol cell responses. Because parasol cells are thought to underlie motion vision in macaques and humans, the observed similarity also suggests that measurements of responses in the front-end visual system in both cats and macaques can be used to make valid predictions for motion vision in humans.

### 4.6. Psychophysical Assumptions

Finally, we considered the factors affecting psychophysical estimates of temporal limits in motion perception. The results presented here are conditional on the definition of the stimulus duration and the psychometric threshold. Moving stimuli were shown in a Gaussian temporal envelope, whose duration is, in theory, infinite. In practice, duration is typically defined as 2σ of the temporal Gaussian (cf. [40]), which includes 68% of total stimulus contrast. The detection threshold was conservatively defined as 82% correct, a commonly used optimal choice for a QUEST staircase [35]. Although our definition of the stimulus duration and selection of the threshold level follow established conventions, they are arbitrary. When tested, the use of other conventions resulted in small changes in duration threshold that did not affect our main findings.

The psychophysical threshold can be affected by inadvertent slips of the subject’s attention, especially for very brief stimuli. To prevent this, the delay between button press and stimulus onset was fixed and therefore predictable for the subjects, who were experienced at the psychophysical task. The use of adaptable staircases to measure thresholds further minimized any possible effects of inattention. The remaining factors influencing psychophysical results are the task and the stimulus parameters. Here, the task was the simplest possible discrimination task. We used periodic stimuli, with stimulus parameters optimized for human motion perception [40] and designed to avoid known inhibitory effects of large moving stimuli [32]. How our results relate to detection of more natural movement patterns such as edge-, contour-, and texture motion is an empirical question that is of interest, and can be addressed in principle using the same experimental approach.

Without direct physiological measurements of the neural correlate of the motion detector’s integrator, we can only infer the exact quantitative relationship between the temporal fidelity of the retina’s output and the temporal limits of motion vision. Instead, we report here for closely matched stimulus conditions, strong similarity and co-dependency on stimulus parameters between predicted optimal integration times and human duration thresholds. Our findings support the hypothesis that the temporal limits of motion vision approximate the limits set by motion encoding in the retina.
